# Unravelling the Effects of Soluble Dietary Fibre Supplementation on Energy Intake and Perceived Satiety in Healthy Adults: Evidence from Systematic Review and Meta-Analysis of Randomised-Controlled Trials

**DOI:** 10.3390/foods8010015

**Published:** 2019-01-06

**Authors:** Siti Nurshabani Salleh, Ahmad Adli Hamizi Fairus, Mohd Nizam Zahary, Naresh Bhaskar Raj, Abbe Maleyki Mhd Jalil

**Affiliations:** 1 School of Nutrition and Dietetics, Faculty of Health Sciences, Universiti Sultan Zainal Abidin, Kuala Nerus 21300, Malaysia; sitinurshabani@gmail.com; 2 School of Biomedicine, Faculty of Health Sciences, Universiti Sultan Zainal Abidin, Kuala Nerus 21300, Malaysia; ahmad.adli95@yahoo.com (A.A.H.F.); nizamzahary@unisza.edu.my (M.N.Z.); 3 School of Rehabilitation Science, Faculty of Health Sciences, Universiti Sultan Zainal Abidin, Kuala Nerus 21300, Malaysia; bnaresh@unisza.edu.my

**Keywords:** soluble dietary fibre, guar gum, alginate, β-glucan, polydextrose, pectin, satiety, energy intake

## Abstract

Consumption of soluble dietary fibre is recommended as part of a healthy diet. Evidence has shown that soluble dietary fibre slows gastric emptying, increases perceived satiety and plays a significant role in appetite regulation. This systematic review examined the effects of soluble dietary fibre using randomised-controlled trials (RCTs). Three different electronic databases were used, namely PubMed, Scopus^®^ and the Cochrane Central Register of Controlled Trials (CENTRAL). Effect size (Cohen’s *d*) was calculated based on the intergroup mean difference and standard deviation (*SD*) followed by Cochran’s Q and *I*^2^ determination. The effect size was statistically pooled in the meta-analyses and presented as a forest plot. The risk of bias was high for each study as assessed using the Jadad scale. Meta-analysis of statistically pooled data for guar gum showed a sizeable effect on post-meal energy intake, followed by β-glucan, alginate, polydextrose and pectin, with pooled effect sizes of −0.90, −0.44, −0.42, −0.36 and −0.26, respectively. Guar gum (5 g) effectively reduced energy intake when prepared in milk beverages compared with control milk (*p* < 0.001). Alginate, when prepared in liquid (5 g) or solid (9 g) meals, effectively reduced energy intake compared with control (*p* < 0.001). A high dose of polydextrose (25 g) prepared in liquid meal form significantly reduced energy intake (*p* = 0.01). This study suggests that soluble fibres are not all created equal. Further interventional studies are needed to determine whether combinations of these soluble fibres might have greater effects than individual fibres per se.

## 1. Introduction

The benefit of dietary fibre on gut health is well established [1]. Dietary fibre is categorised into soluble and insoluble fibre. The distinction between soluble and insoluble dietary fibres is that the former solubilise in hot aqueous solution [2]. The physiological effects of soluble dietary fibres are attributed to its unique physico-chemical properties, namely viscosity, gel formation or fermentability in the colon [3]. Previous studies have demonstrated the physiological effects of soluble fibre as follows: (1) increased viscosity in the upper gastrointestinal tract [4,5], and (2) partial or full fermentation in the colon [6] and (3) exertion of a prebiotic effect [7,8].

Different dietary fibres might have different viscosities depending on their structure, concentration and chemical composition (types of monomers) [9,10]. Viscous soluble dietary fibres are believed to be more capable of inducing satiety compared to non-viscous soluble dietary fibres. Viscous soluble dietary fibre increases digesta viscosity and consequently delays gastric emptying [11,12,13], slowing digestion and the absorption of nutrients [14], and reducing enzyme diffusion [15] and the formation of an unstirred water layer [16]. Viscous soluble dietary fibres are not being digested in the stomach; instead, they are fermented in the colon and result in a rise in short chain fatty acids (SCFAs). SCFAs, particularly propionate, modulate the secretion of various appetite-regulating peptides (PYY, GLP-1 and CCK) throughout the colon and are associated with increased perceived satiety and reduced food intake [17,18,19,20]. Furthermore, the presence of soluble dietary fibre selectively boosts the growth or activity of a restricted number of colonic bacteria, ultimately enhancing host health [21]. Soluble fibre alters the balance of the gut microflora towards what is considered to be a healthier one [22].

Satiety is defined as a process that leads to inhibition of further eating, declining hunger and an increase in fullness after a meal, leaving one satisfied [23]. However, there have been inconsistent findings regarding the benefits of soluble dietary fibre on perceived satiety. Studies have shown the effects of soluble fibre to depend on factors such as dose, molecular size and solubility, and food matrix [24,25]. Hence, the aim of this review was to answer the following questions: (1) What is the best type of soluble dietary fibre to reduce energy intake? (2) What is the best dose for such an effect? (3) What is the optimal food matrix (solid, semi-solid or liquid meal)? The information was extracted from randomised-controlled trials followed by qualitative and quantitative analyses.

## 2. Methods

### 2.1. Eligibility Criteria

This review included studies examining healthy male or female free-living adults aged 18 years or above with a normal body mass index (18.5 to 24.9 kg/m^2^) [26] and not taking any supplements or antibiotics during the study period. The review excluded studies in which participants had undergone surgical procedures with the potential to affect gastrointestinal function or digestibility, which may interfere with results and contribute to clinical heterogeneity [27].

This review examined only interventions investigating the effects of soluble dietary fibre using randomised-controlled trials (RCTs). RCTs were chosen as they are considered as cornerstone of clinical research on intervention and offer the highest level of evidence [28]. Studies were excluded if the comparator groups were absent or not specified [29]. The outcome measures are as follows: energy intake, perceived satiety, appetite hormones, gastric emptying time and colonic transit time. Studies were excluded if they were observational in nature, that is, cross-sectional, retrospective or prospective cohort, longitudinal or case-control studies as well as case reports, case series, animal or in vitrostudies. There was no restriction on the time frame and type of study setting. Only full-text English articles were included in this review. Studies written in languages other than English were excluded due to potential bias of information resulting from poor translation. Given advances in research methodology, only studies published between 2007 and 2017 were included.

### 2.2. Search Strategy

Three different electronic databases were used to systematically search the literature as follows: (i) PubMed website (US National Library of Medicine and National Institute of Health); (ii) Scopus^®^ (Elsevier B.V.); and iii. The Cochrane Central Register of Controlled Trials (CENTRAL, Cochrane library). PubMed was selected as it contains 27 million biomedical studies from MEDLINE and life science journals. Scopus was chosen as it is the largest database for scientific journals covering the fields of science and medicine, with 100% MEDLINE, EMBASE and Compendex. The Cochrane Central Register of Controlled Trials (CENTRAL) is the largest database and the most comprehensive source of reporting for RCTs. The search strategy for all three databases was carried out over the course of two weeks in August 2017. For Scopus, only articles in the fields of ‘agriculture and biological science’, ‘biochemistry’, ‘genetics and molecular biology’ and ‘medicine’ were included.

Boolean operators were included in the keyword searches of all three electronic databases. The main keywords were ‘type of soluble fibre’ AND ‘appetite’ AND ‘satiety’. Key search terms for type of soluble fibre were ‘soluble dietary fibre’, ‘soluble dietary fiber’, ‘beta glucan OR beta glucans OR b-glucan OR β-glucan’, ‘guar gum OR guaran OR gellan gum’, ‘alginate OR alginates OR alginic OR algin’, ‘pectin OR pectins’, ‘laminarin OR laminarins’, ‘polydextrose’, ‘PolyGlycopleX’, ‘maize-based soluble fibre OR maize-based soluble fiber’, ‘galactomannan OR galactomannans/konjac’, ‘NUTRIOSE’, ‘plantain’, ‘soluble corn fiber OR fibre’, ‘soluble maize fiber OR fibre’, ‘arabinoxylan OR arabinoxylans’, ‘mixed-linkage glucans’, ‘pullulan’, ‘plant gum OR plant gums OR gum OR gums OR plant mucilage OR mucilage’. Key search terms for appetite and satiety were ‘appetite hormones’, ‘gut hormones’, ‘glucagon-like peptide 1 OR GLP-1′, ‘Peptide YY OR PYY’, ‘cholecystokinin OR CCK’, ‘gastric insulinotropic polypeptide OR GIP’, ‘colonic transit time’, ‘gastrointestinal’, ‘gastric emptying time’, ‘energy intake OR energy intakes’ and ‘satiety’.

### 2.3. Data Management and Analysis

All articles were uploaded in Mendeley referencing software and duplication removed use of the ‘remove duplicate’ function. Three reviewers independently screened the titles and abstracts based on the pre-defined criteria described above. Full-text articles were carefully reviewed to determine whether the articles met the inclusion or exclusion criteria. Primary data extraction was done to identify studies investigating the effects of soluble fibre on energy intake, perceived satiety, appetite or gut hormones, gastric half-emptying time and/or colonic transit time as outcome measures. Secondary data extraction was done to exclude studies that did not meet the defined criteria, studies that failed to report certain details and studies that did not fall within the definition of RCTs. Data extraction included RCTs, the type of comparator clearly described (i.e., control/placebo), sample size calculation, dosage used, study duration, participant characteristics (free-living, normal body mass index (BMI) and healthy) and type of soluble dietary fibre related to outcome of interest.

### 2.4. Evaluation of Studies and Data Synthesis

For primary outcomes, the effect size (Cohen’s *d*) was calculated based on the intergroup mean difference and standard deviation (*SD*) in energy intake, perceived satiety, gastric half-emptying time, and appetite or gut hormones between treatment and control groups. The quantitative measure of the standardised mean differences (effect sizes) between the groups was considered as small (0.2), medium (0.5) and large (0.8). A negative value indicates that the intervention favoured the treatment group, while a positive value indicates that the intervention favoured the control or placebo group [30]. Means and standard deviations were manually calculated when the exact outcomes measures were not reported. The standard error of the means was converted into standard deviations to derive Cohen’s *d* [31]. *P-values* of less than 0.05 were deemed statistically significant. A study was excluded from the meta-analysis if the effect size could not be calculated.

For the secondary outcomes, Cochran’s Q and *I*^2^ were manually calculated using Excel worksheets in accordance with Neyeloff, Fuchs, and Moreira [32]. Cochran’s Q was calculated as the weighted sum of squared difference between each study’s effects and the pooled effect across studies with the weight used in the pooling method. Q was distributed as a chi-square statistic (*χ*^2^) with *k* minus 1 degree of freedom (df) whereas *k* is number of studies. The *p*-value was obtained by comparing Q against a table of critical values where a lower Q indicated that the studies were similar (i.e.,homogenous). The *I*^2^ statistic describes the percentage of total variation across studies due to true heterogeneity. *I*^2^ was calculated based on the formula (Q−df)/Q × 100, where ‘df’ stands for degrees of freedom and Q is Cochran’s heterogeneity statistic. A negative value of *I*^2^ was considered to be zero (studies were homogenous). Statistical heterogeneity of meta-analyses (*I*^2^) values of 25%, 50% and 75% were considered as low, moderate and high, respectively [33]. A fixed-effect model was selected if the test of heterogeneity was not significant and the *I*^2^ value was low (< 50%). The random effects model was used for heterogeneity ≥ 50%. The effect size was statistically pooled in the meta-analyses and presented as a forest plot.

The risk of bias of each individual study was assessed using the Jadad scale. The scale specifies whether double-blinding, randomisation, drop-outs and withdrawals were clearly described in the study [34]. The highest possible score is 5 and is suggestive of a low potential for reporting bias. Studies were rated as having low, moderate or high risk of bias.

## 3. Results

### 3.1. Study Selection

**Figure 1:**
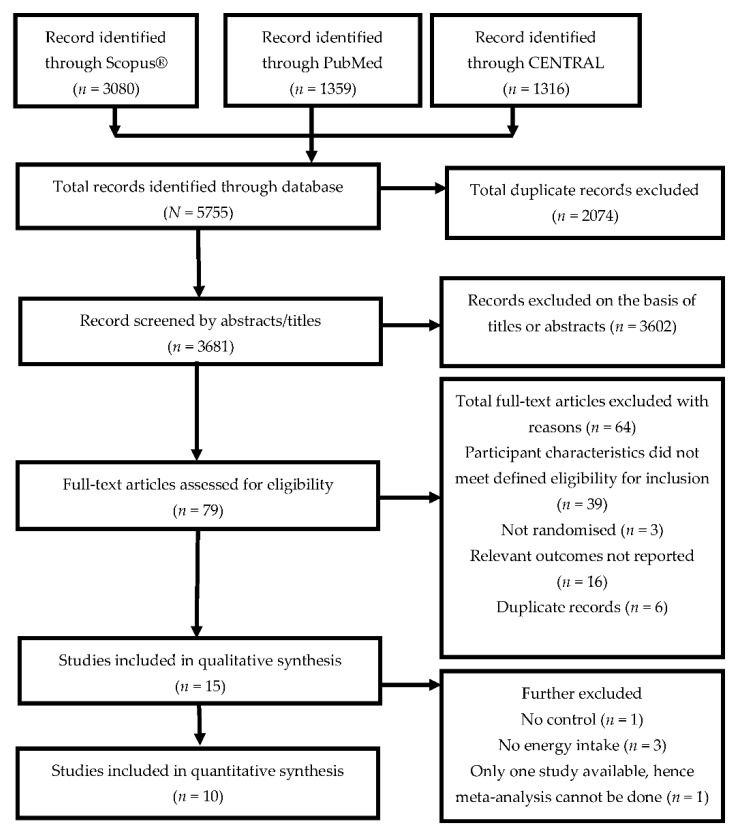
PRISMA flow chart of search strategy.

Figure 1 illustrates the study selection based on the PRISMA search strategy. An initial sample of 5755 articles was identified. Of these, 3080 articles were from Scopus^®^, 1359 articles were from PubMed, and the remaining 1316 articles were from CENTRAL. Duplicates were excluded and all articles were screened on the basis of their titles and abstracts. Seventy-nine full-text articles were retrieved to assess their eligibility, and 15 articles met the defined criteria and were subsequently included in the qualitative analysis. A subset of 10 articles was included in the meta-analysis.

### 3.2. Study Characteristics

Table 1 presents a summary of the randomised-controlled trials (RCTs) (*N* = 15 articles) involving 17 interventional studies with 31 soluble fibres [35,36,37,38,39,40,41,42,43,44,45,46,47,48,49]. A total of eight soluble dietary fibres were identified: alginate, arabinoxylan, β-glucans, guar gum, high-amylose maize, pectin, polydextrose, and PolyGlycopleX. There were a total of 31 soluble fibre types, doses or viscosities included in this review as follows: alginate (*n* = 3), arabinoxylan (*n* = 2), β-glucans (*n* = 3), guar gum (*n* = 6), high-amylose maize (*n* = 2), pectin (*n* = 4), polydextrose (*n* = 8) and PolyGlycopleX (*n* = 3). While all 31 soluble fibres were included in the systematic review (qualitative study), only 21 were included in the meta-analysis (quantitative study). Ten fibres were excluded from the meta-analysis for the following reasons: energy intake not measured (*n* = *7*), control not available (*n* = 1) and only one study available, hence meta-analysis cannot be done (*n*= 2).

The doses of fibre used in the studies were in the range of 2 to 31.5 g and supplemented either liquid, semi-solid, solid or composite meals. All studies included were of randomised crossover-controlled design. Of the 17 interventional studies, one was double-blinded, 13 were single-blinded and three were not-blinded. The sample size ranges from 6 to 121 participants with a total sample size of 453 participants. The durations of outcome measures for energy intake, satiety, gut hormones and gastric emptying time range from 90 to 240 min, 30 to 240 min, 90 to 180 min and 90 to 360 min, respectively. The shortest duration of food intake measures was 90 min [42,44], while the longest was 240 min [38,41] (Table 1).

### 3.3. Outcome Measures

**Figure 2:**
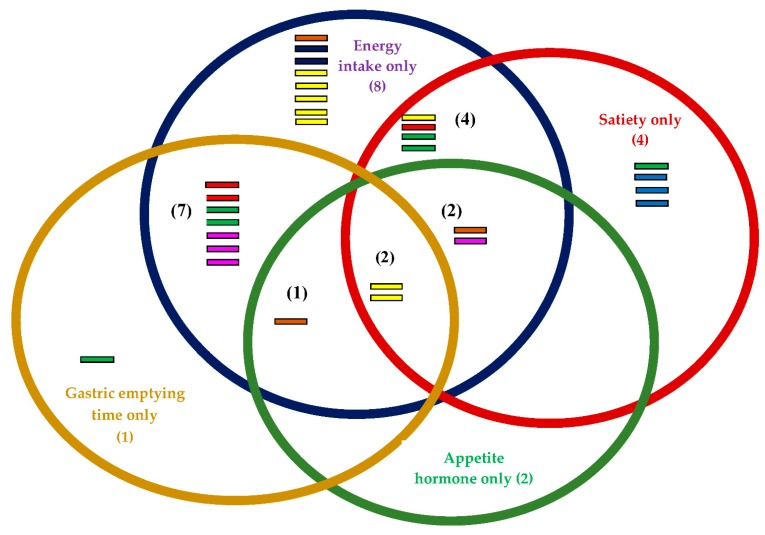
Outcome measure for each fibre: Energy intake (blue circle) = 24; Satiety (red circle) = 12; Gastric emptying time (yellow circle) = 11; Appetite hormone (green circle) = 7; 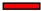
Alginate
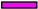
Pectin
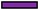
Arabinoxylan
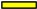
Polydextrose
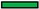
Guar gum
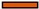
β-Glucan
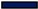
High amylose maize
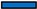
Polydextrose.

The outcome measures in this review are energy intake, perceived satiety, appetite hormones and gastric emptying time. Twenty-four of the 31 studies investigated the effects of soluble fibre on energy intake between treatment and control groups [35,36,37,38,39,40,41,42,43,44,45,48]. Twenty-two studies favoured treatment, but only four reported significant differences [34,36,37,38,39,40,41,42,44,45,46,47,48] (Table 1). Another 12 of the 31 studies focused on satiety, while 11 out of 31 investigated the gastric emptying rate and seven out of 31 investigated the effects of soluble dietary fibre on appetite hormones (PYY, GLP-1, CCK and GIP) (Figure 2).

It was observed that only four test products significantly reduced energy intake, as follows: 5 g of alginate and 5 g of guar gum in milk beverages [35], 9 g of alginate in chocolate cookies [36] and 25 g of polydextrose in chocolate-flavoured beverages [42] (Table 1). However, all fibres supplemented in the liquid food matrix favoured treatment rather than control groups [35,37,39,40,42,44]. Soluble fibre supplemented in the solid food matrix such as cookies, soya bean curd, white wheat bread or cooked white rice did not significantly reduce energy intake, except for high-dose alginate in chocolate cookies [36]. B-glucan (2.9 g) [38] showed a higher effect size on energy intake reduction compared with 6 g of alginate [36], with *d* = −0.35 (*p* = 0.28) (medium effect size) and *d* = −0.04 (*p* = 0.78) (small effect size), respectively. A high dose of alginate (9 g) is necessary in order to have significant effects (effect size = −0.52, *p* < 0.001) on reduced energy intake [36]. However, the effect of 9 g of alginate supplemented in chocolate cookies was larger than the effect of 12 g of polydextrose added to low- and high-protein soya bean curd, with effect sizes of −0.42 and −0.48, respectively [43].

Juvonen et al. [45] demonstrated that high viscosity β-glucan reduced energy intake compared with low viscosity β-glucan, with an effect size of −2.03 (*p* = 0.026). Wanders et al. [40] investigated the effects of 10 g of pectin in different forms, that is, bulking, viscous and gelled, on energy intake. No significant effects were observed with different types of pectin on energy intake compared with control (Table 1). Gelled pectin was further supplemented in liquid (as beverage) and capsule (so the pectin hydrated in the stomach) form. Pectin in capsule form exhibited lower energy intake than pectin in liquid form (*p* = 0.03) (Table 1). The lowest dose of soluble fibre found in this systematic review was 2 g of partially hydrolysed guar gum [37], while the highest dose was 31.0 g of high-amylose maize [48]. However, 2 g of partially hydrolysed guar gum showed a higher effect size on energy reduction (*d* = −0.41, *p* = 0.28) than 31.0 g of high‒amylose maize (*d* = −0.05, *p* = 0.83).

### 3.4. Risk of Bias within Studies Based on Jadad Score

Table 2 illustrates the quantified risks of bias within a study according to the features of RCTs, namely randomisation, double-blinding and withdrawals or drop-outs [34]. Risks of bias were relatively high with most studies showing an average score of 3 and below [35,36,37,38,39,40,41,42,43,44,45,46,47,48,49]. Five studies showed moderate risk of bias [37,40,42,43,49] while a further 12 showed high study bias [35,36,38,39,45,46,47] (Table 2).

### 3.5. Random Effects Analysis

**Figure 3:**
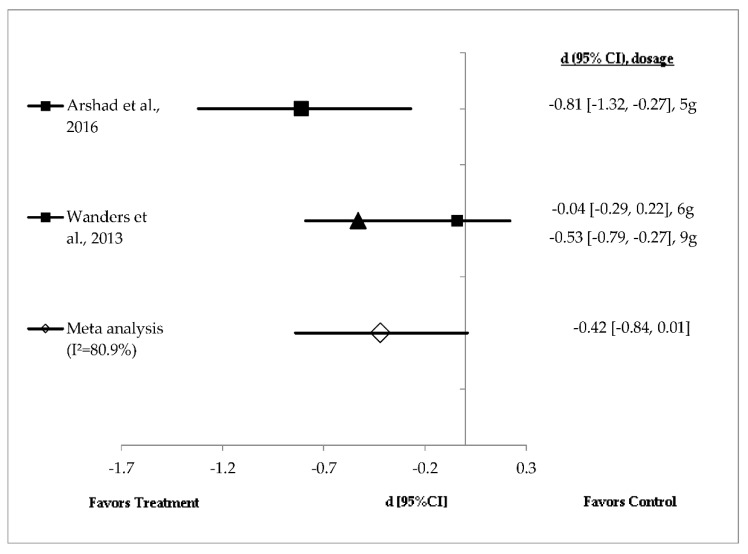
Effects of alginate on energy intake reduction (random effects model). The axis lines on ±0.2, ±0.5 and ±0.8 represent small, medium and large effect sizes, respectively. A negative value of summary effect size suggests that the alginate supplementation decreases energy intake compared with control. ▲: 9 g alginate; ■: 6 g alginate.

**Figure 4:**
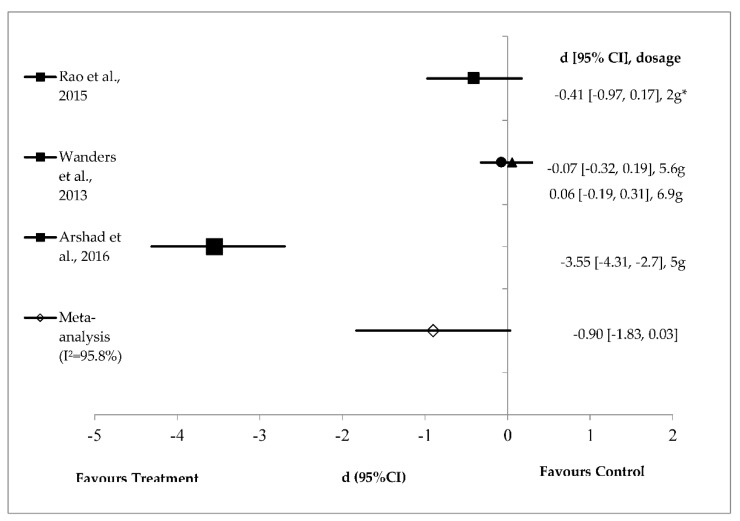
Effects of guar gum on energy intake reduction (random effects model). The axis lines on ±0.2, ±0.5 and ±0.8 represent small, medium and large effect sizes respectively. A negative value of summary effect size suggests that the guar gum supplementation decreases energy intake compared to control. *2 g of partially hydrolysed guar gum (PHGG); ●: 5.6 g of guar gum; ▲: 6.9 g of guar gum.

Studies investigating the effects of alginate (*n* = 2) and guar gum (*n* = 3) on energy intake showed a high level of heterogeneity and hence were analysed using random effects analysis (Figure 3 and Figure 4). Figure 3 shows the effect sizes of mean energy intake reduction in studies with alginate supplementation (*n* = 2), ranging from very small (*d* = −0.04, *p* = 0.78) to large (*d* = −0.81, *p* < 0.001). Mean energy intake reduction in all three doses of test product favoured treatment. Five grams of alginate in milk beverages (liquid) and 9 g in chocolate cookies (solid) significantly (*p* < 0.05) reduced energy intake compared with control [35,36]. The effect size of 5 g of alginate was larger [35] than that of 9 g of alginate [36]. However, a meta-analysis showed a medium and non-significant difference in mean energy intake reduction favouring alginate supplementation (pooled effect size of −0.42; 95% CI (–0.84, 0.01); *I*^2^ = 80.9%) (Figure 3).

Figure 4 shows the effect size of mean energy intake reduction after guar gum supplementation (*n* = 3). The effect sizes range from small (*d* = 0.06, *p* = 0.65) to very large (*d* = −3.55, *p* < 0.001). Mean energy intake in three test products favoured treatment groups except for 6.9 g of guar gum [36]. Either 2 g of partially hydrolysed guar gum or 5 g of guar gum prepared in the liquid food matrix significantly reduced energy intake [35,37]. However, a large but non-significant pooled effect size of −0.90 (95% CI (−1.83, 0.03); *I*^2^ = 95.8%) was observed for guar gum on energy intake reduction (Figure 4).

### 3.6. Fixed Effects Analysis

**Figure 5:**
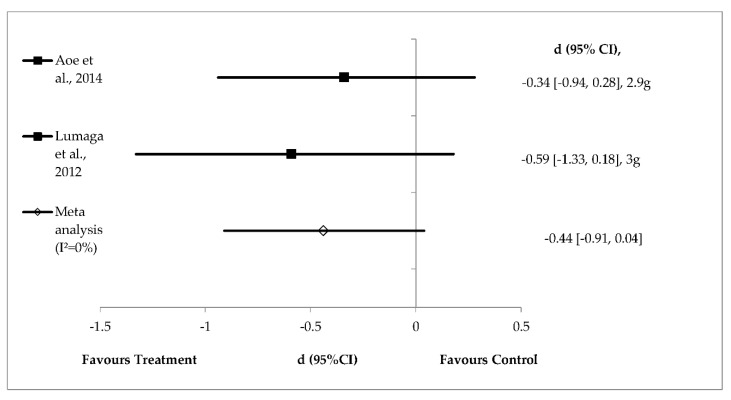
Effects of β-glucan on energy intake reduction (fixed effects model). The axis lines on ±0.2, ±0.5 and ±0.8 represent small, medium and large effect sizes, respectively. A negative value of summary effect size suggests that β-glucan supplementation decreases energy intake compared with control. *d* = effect size; CI = confidence interval.

**Figure 6:**
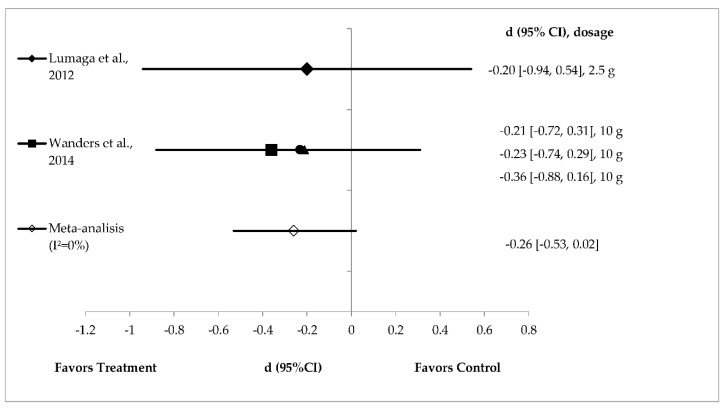
Effects of pectin on energy intake reduction (fixed effects model). The axis lines on ±0.2, ±0.5 and ±0.8 represent small, medium and large effect sizes respectively. A negative value of summary effect size suggests that pectin supplementation decreases energy intake compared to control. *d* = effect size; CI = confidence interval. ▲: bulking; ■: gelled; ●: viscous.

**Figure 7:**
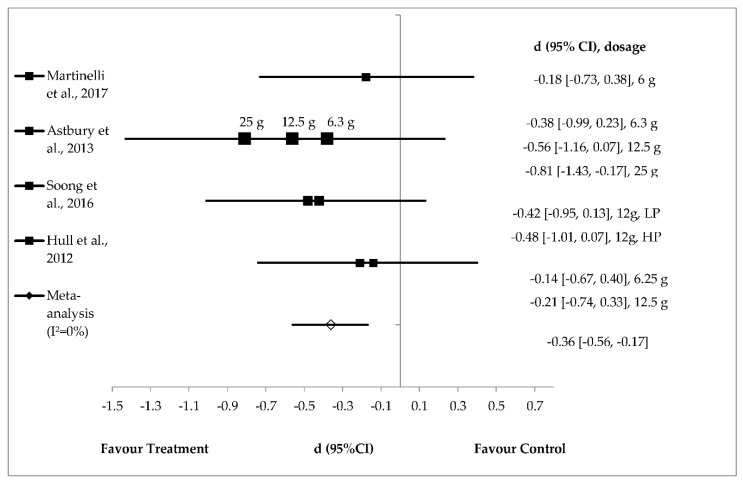
Effects of polydextrose on energy intake reduction (fixed effects model). The axis lines on ±0.2, ±0.5 and ±0.8 represents small, medium and large effect sizes respectively. A negative value of summary effect size suggests that polydextrose supplementation decreases energy intake compared to control. d = effect size; CI = confidence interval; LP = low protein; HP = high protein.

The level of heterogeneity was low for the effects of β-glucan (*n* = 2), pectin (*n* = 2) and polydextrose (*n* = 4) on energy intake and hence these were analysed using fixed effects analysis (Figure 5, Figure 6 and Figure 7). Meta-analysis of the studies shows a non‒significant difference in mean energy intake reduction favouring β-glucan supplementation with medium effect (pooled effect size of −0.44; 95% confidence interval: −0.91 to 0.04; *I*^2^ = 0%). Individual studies of β-glucan showed high effect size when prepared in liquid (*d* = −0.59, *p* = 0.13) compared with solid meal form (*d* = −0.34, *p* = 0.28) [38,39] (Figure 5).

Pectin supplementation (*n* = 2) did not significantly reduce energy intake with effect sizes of −0.20 to −0.36 [39,40]. A small and non‒significant pooled effect size of −0.26 (95% CI (−0.53, 0.02); *I*^2^ = 0%) was observed for pectin on energy intake reduction (Figure 6).

Polydextrose supplementation (*n* = 4) resulted in a small (−0.14) to a large (−0.81) effect size on mean energy intake reduction [41,42,43,44] (Figure 7) with a small and significant pooled effect size of 0.36 (95% CI (−0.56, −0.017); *I*^2^ = 0%) (Figure 7). Polydextrose supplemented in chocolate-flavoured beverages significantly reduced energy intake with a large effect size of ‒0.81 [42]. Polydextrose (6.25 g) prepared in liquid matrix and polydextrose (6 g) in pudding with semi-skimmed milk (composite meal) showed small effect sizes of *d* = −0.14 and *d* = −0.18, respectively. Interestingly, this review also found that polydextrose with the same doses (12.5 g) and same liquid food matrix exhibited distinct effect sizes, both small (*d* = −0.21) and medium (*d* = −0.56) [42,44] with small and significant pooled effect size of −0.36 (95% CI (−0.56, −0.17); *I*^2^ = 0%) (Figure 7).

## 4. Discussion

A total of eight soluble fibres were identified as follows: alginate, guar gum, arabinoxylan, β-glucan, high-amylose maize, pectin, polydextrose and PolyGlycopleX. Guar gum showed the greatest energy intake reduction, followed by β-glucan, alginate, polydextrose and pectin. The risk of bias was high; only four studies clearly reported how randomisation was conducted. The randomisation procedure was not clearly described in most studies, resulting low Jadad scores. Only one study was double-blinded [37].

The pooled random effects analysis showed that alginate did not significantly reduce energy intake in healthy adults (Figure 3). However, when considering individual studies, 5 g of alginate prepared in 250 mL milk beverages showed a larger and significant effect (*d* = −0.81, *p* < 0.001) on energy intake reduction than 6 g alginate in chocolate cookies (small effect size: *d* = −0.04, *p* = 0.78) [35,36]. The time interval for energy intake after consumption of 6 g alginate in cookies was shorter compared with alginate in liquid drinks, at 105 and 120 min, respectively. It is expected that a shorter time interval between fibre and ad libitum energy intake would decrease food intake. Hence, it is possible that other factors, such as preloads energy density (kcal/g) and dose may be responsible for such an effect. A higher dose of alginate (9 g) in the solid food matrix increased oral processing time and reduced the rate of gastric emptying [36]. Mechanistically, alginate may form a gel at low pH or in the presence of divalent cations [50]. Alginate forms a gel in the oral cavity due to the presence of water and divalent cations from saliva [51]. The measurement of physico-chemical properties suggests that alginate increases water‒holding capacity as well as digesta viscosity [3,36].

Random effects analysis showed that guar gum (*n* = 3) did not significantly reduce energy intake in healthy adults (Figure 4). However, when considering individual studies, guar gum (5 g) in milk beverages showed a large and significant effect on energy intake (*d* = −3.55, *p* < 0.001) [36]. The preparation of guar gum in milk beverages reduced short-term food intake and satiety (120 min) compared with control preloads [36]. This effect might be due the formation of a more stabilised gel emulsion between milk protein and guar gum in the stomach [52]. Other study has shown reduced hunger ratings with increasing beverage viscosity [53]. However, the effect might be different when guar gum is added to the solid food matrix. Wanders et al. [36] showed that both 5.6 g and 6.9 g of guar gum prepared in cookies did not reduce ad libitumfood intake compared with control. The study suggested that the liquid food matrix showed more pronounced effects on reduced energy intake compared with solid food matrix. Rao et al. [37] showed that long-term (two-week long) intake of 2 g partially hydrolysed guar gum (PHGG) for 14 days significantly reduced energy intake at lunch and evening snacks compared with control dextrin. PHGG is produced from guar gum with the same molecular structure but with shorter chain length [54]. Natural guar gum is extremely viscous and might form a very viscous product when added to products, lowering acceptability. Hence, a low molecular weight guar gum is more favourable than a high molecular weight one for product development. An early study by Ellis et al. [55] showed that low molecular weight guar gum markedly increased product palatability compared with high molecular weight guar gum.

Pectin is a natural fibre present in fruit and vegetables and one of the major plant cell wall components. Pectin has varying viscosity and gelling ability in accordance with its molecular weight [56]. Pectin behaves differently when hydrated in the liquid food matrix [40]. Wanders et al. [40] showed that viscous (80 kDa) and bulking pectin (25 kDa) had similar effects on energy reduction followed by gelled pectin (15 kDa). However, the effects were not statistically significant [40]. This study demonstrated that 10 g of pectin with a molecular weight of 15 kDa formed a gel while 80 kDa pectin increased the viscosity of dairy-based liquid drinks. All three type of pectin reduced gastric emptying, as indicated by ^13^C recovery in breath samples. However, this study showed that reduced gastric emptying was not associated with a reduction in energy intake. Wanders et al. [40] further investigated different modes of pectin delivery, namely supplemented as equal dose of gelled pectin (10 g, 15 kDa) in liquid beverage and capsule form. The latter approach was taken so that the pectin would form a gel in the stomach. The encapsulated pectin significantly reduced energy intake compared with gelled pectin in a liquid beverage [40]. Product viscosity rather than stomach viscosity reduced ad libitum intake [57]. It may be that the encapsulated pectin is entrapped in the food matrix within the stomach and must be hydrated first [3,58]. More water was retained, increasing small bowel transit time [59,60].

β-glucan is a soluble fibre extracted from oat and barley. Four grams of β-glucan per 30 g of available carbohydrate has been approved by the European Food Safety Authority to reduce blood glucose without disproportionately increase insulin levels [61]. β-glucan confers other health benefits, such as improving insulin resistance, dyslipidaemia, hypertension and obesity [62]. The results from fixed effects analysis show that β-glucan does not significantly reduce energy intake (Figure 5). In this review, 2.9 g of β-glucan in the solid food matrix (cooked white rice) did not reduce energy intake compared with control (*d* = −0.34, *p* = 0.28) [38]. β-glucan supplementation (3.0 g) in beverages reduced energy intake (medium effect size, *d* = −0.59), but the difference was not significant (*p* = 0.13) compared with control [39]. These two individual studies suggest that 2.9 g to 3.0 g of β-glucan either in the liquid or solid food matrix had small to medium, non-significant effects size on reducing energy intake. The satiety mechanism is complex and involves an integrated physiological system (the food-gut-brain axis) [63]. These physiological responses determine what we eat and how much of it we consume. In this review, we focused on the role of adding soluble fibre to food to manipulate the satiety response. Research has shown that adjusting the energy density of food by increasing its volume (e.g., by adding water or dietary fibre) while maintaining the macronutrient composition might be a good strategy to enhance satiety [64].

The effect of β-glucan on perceived satiety depends on factors such as dose, molecular weight and solubility, and food matrix. In the context of doses, this review found that 3.0 g of β-glucan supplemented in 250 mL beverages significantly increased satiety compared to control beverages [39]. This finding was in line with Lyly et al. [24] who reported that 2.5 g of β-glucan in 300 mL beverages significantly increased perceived satiety compared with fibre-free beverages. The physical effects of β-glucan on the ingesta appear to be fundamentally important in shaping their satiating properties. This effect is highly dependent on the molecular size and solubility of β-glucans [25]. The molecular weight of β-glucans varies from 31 to 3100 kDa and is a major determinant of their solubility in water and, hence, satiety [65]. The molecular weight of β-glucan varies depending on the production process, that is, isolation, purification, and extraction [66].

Food matrix might also play a role on the effect of β-glucan and satiety. In theory, solid foods are more satiating than liquid foods [67]. However, most studies have failed to show any significant effect of β-glucan on satiety when prepared in solid or semi-solid compared with liquid meals [24]. Soluble fibres, when prepared in liquid meal form, absorb more water and increase stomach distension, triggering afferent vagal signals to stop eating (i.e., increasing fullness) [68]. Mattes and Rothacker [53] elegantly demonstrated that a higher viscosity shake (16,000 cps) was more effective at reducing perceived hunger than a low viscosity shake (600 cps). Lyly et al. [69] showed that beverages enriched with soluble β-glucan increased perceived satiety compared with fibre-free beverage. However, both beverages (β-glucan and control) showed lower perceived satiety compared with a solid meal (white bread). This suggests that the presence of both liquid and solid food within a trial might mask the satiating potential of β-glucan. A similar study has demonstrated that the more pronounced satiating effect of solid food per se may ‘mask’ the satiating potential of β-glucan [62]. Juvonenet al. [45] demonstrated that increased viscosity produced by oat β-glucan in liquid meal markedly reduced postprandial appetite hormones, namely, CCK, PYY and GLP-1. High-viscosity β-glucan drinks reduce intestinal mixing and might prevent the interaction between the nutrients and enteroendocrine L cells in the distal colon for appetite hormones release [70]. Three-hour ad libitum energy intake was similar between low- and high-viscosity beverages but was significantly lower when energy intake was combined for the rest of the day [45]. However, it must be noted that appetite hormones do not necessarily correlate with food intake and vary between individuals [71]. Lumaga et al. [39] demonstrated that β-glucan in liquid beverages did not reduce energy intake or significantly affect appetite hormones (PYY, GLP-1 and GIP) but did significantly reduce perceived satiety compared with the control. One study showed that the average colonic transit time for the Western population is 30–40 h [72]. Other studies demonstrated that the effects on appetite might take up to 6 h after the ingestion of soluble fibre [73,74]. Based on this evidence, it is highly advisable to consider the time frame for the measurement of appetite hormones.

In this review, we focused on four main outcome measures namely gastric emptying time, appetite hormones, energy intake and perceived satiety (Figure 2). The majority of the studies focused on measuring energy intake in isolation (*n* = 8) and energy intake plus gastric emptying time (*n* = 7). Only two studies (*n* = 2, both are polydextrose) measured all four outcomes. It is strongly advisable to measure all four elements to have a better understanding of the effects of dietary fibre on appetite regulation. Polydextrose is a type of soluble dietary fibre and is not digested in the upper gastro-intestinal tract. Instead, it is partially fermented in the large intestine for the production of SCFAs [75]. Polydextrose is resistant to digestion and is partially fermented by the intestinal microbiota [76,77]. Polydextrose is well tolerated up to a dose of 90 g/day and has no laxative effect [78]. In this review, the heterogeneity for polydextrose was low (*I*^2^ = 0%), and these studies were, therefore, analysed using fixed effects analysis. Polydextrose showed a medium-sized pooled effect (*d* = −0.36, *p* < 0.05) on energy intake reduction. An individual study showed that 25 g of polydextrose significantly reduced (*d* = −0.81, *p* < 0.001) energy intake (90-min post preload) when prepared in 400 mL chocolate-flavoured liquid drinks compared with control [42]. This was consistent with the findings of the previous study, which demonstrated that 25 g of polydextrose in 200 g of yogurt significantly reduced energy intake compared with control yogurt. Similar to other soluble fibres, polydextrose increases gastrointestinal viscosity and may be subject to colonic fermentation for the production of SCFA [79,80,81]. However, lower doses of 6.25, 6.3 and 12.5 g of polydextrose prepared in liquid beverages were less effective at reducing energy intake [42,44]. Soong et al. [43] showed that 12 g of polydextrose prepared with low- and high-protein (LPP and HPP) soya bean curd significantly reduced gastric emptying time compared with the control. However, the supplementation did not reduce energy intake or increase perceived satiety compared with the control. LPP and HPP marginally increased GLP-1 compared with control soya bean curd. This is consistent with the results of other studies which have shown GLP-1 to slow gastric emptying in individuals following a high-fibre diet [82,83].

## 5. Conclusions

In this review, the evidence has shown that soluble fibre can potentially be used as an active ingredient for the formulation of functional foods. Soluble fibres might play a role in reducing energy intake and hence could be incorporated in the daily dietary intake. However, not all soluble fibres have similar effects on appetite regulation, that is, appetite hormones, gastric emptying and perceived satiety. Further long-term study is needed to determine whether these fibres could reduce energy intake and hence help maintain long-term body weight. Future research should aim to determine whether there is a synergistic effect when combining different soluble fibres together in the food matrix to reduce energy intake and/or stimulate appetite hormones. In addition, studies have not focused on the effects of soluble dietary fibre on colonic transit time. Thus, there is a need for future studies to explore this angle. This systematic review suggests that the food matrix of preloads is important to induce satiety, preferably as the liquid food matrix. Based on our earlier research questions, 5 g of guar gum in a liquid meal is optimal for reducing subsequent energy intake. Guar gum effectively reduces energy intake, but it is not always as palatable as other soluble fibres. We suggest using a combination of guar gum with other soluble fibres, for instance alginate, β-glucan, pectin and/or polydextrose, in order to improve palatability, reduce energy intake and increase appetite hormones.

## Figures and Tables

**Table 1:** Summary of randomized-controlled trials (RCTs) included in systematic review (*n* = 15).

Study	Fibre type	Dose	Study Overview	Appetite Hormone (Mean (*SD*)) (pmol·min/L)	Gastric Emptying Rate (min)	Energy Intake (kcal)	Perceived Satiety (Mean (*SD)*) (mm)
Arshad et al., 2016 [35]	Alginate	5 g	Subjects: 30 (F: 18–30 y) Study design: randomised, single blinded Washout: 1-week Energy intake interval: 120 min Satiety: 170 min Food matrix: liquid (milk served chilled and iso-volumetric (250 mL) Control: milk (250 mL) without fibre	Data not available	Data not available	611.76 (35.01) (*p* < 0.001) Effect size = −0.81	76.36 (1.82) (*p* < 0.05) Effect size = −0.90
	Guar gum	5 g		Data not available	Data not available	551.71 (23.52) (*p* < 0.001) Effect size = −3.55	61.81 (1.82) (*p* < 0.05) Effect size = −3.93
Wanders et al., 2013 [36]	Alginate (MW: 60–1000 kDA)	(i) 6 g (ii) 9 g	Study 1 (energy intake): Subjects: 121 (45M/76F: 18–50 y) Study design: randomised, single blinded Washout: 2-day Energy intake interval: 105 min Food matrix: solid (400 g chocolate cookies with 500 mL of water) Control: chocolate cookies without fibre Study 2 (gastric emptying): Subjects: 10 (4F/ 6M: 18–50 y) Study design: randomised, single blinded Washout: 7-day Gastric emptying rate: every 15 min and up to 240 min Food matrix: solid (400 g chocolate cookies with 500 mL of water) Control: chocolate cookies without fibre	Data not available	Study 2: AUC, 13C breath used (i) 2126 (*p* < 0.05) (ii) 2145 (*p* < 0.01) No *SD* available to calculate effect size	Study 1: (i) 943.44 (406.04) (*p* = 0.77) Effect size = −0.04 (ii) 702.68 (429.92) (*p* < 0.001) Effect size = −0.52	Data not available
	Guar gum (MW: 17–710 kDA)	(i) 5.6 g (ii) 6.9 g		Data not available	Study 2: AUC, 13C breath used (i) 1918 (*p* > 0.05) (ii) 1864 (*p* > 0.05) No *SD* available to calculate effect size	Study 1: (i) 931.50 (501.58) (*p* = 0.63) Effect size = −0.07 (ii) 979.27 (525.46) (*p* = 0.66) Effect size = 0.06	Data not available
Rao et al., 2015 [37]	Partially hydrolsed guar gum (PHGG)	Study 1: 2 g PHGG	Study 1 (breakfast, liquid yogurt): Subjects: 24 (12M/ 12F) Study design: randomised, double blinded Washout: 2-weeks Energy intake interval: 180 min Satiety: 240 min Food matrix: liquid (yogurt, 125 g) Control: Energy Intake: yogurt with dextrin Satiety: yogurt without fibre Study 2 (lunch, solid rice): Subjects: 6 (4M/ 2F) Study design: randomised, single blinded Washout: 2-days Satiety: 300 min Food matrix: solid (cooked rice) Control: cooked rice without fibre addition	Data not available	Data not available	Study 1: 731.60 (162.76) (*p* = 0.17) Effect size = −0.41	Study 1 (breakfast, 2 g): 14.9 (15.19) (*p* < 0.05) Effect size = 0.46
		Study 2: 6 g PHGG		Data not available	Data not available	Data not available	Study 2 (lunch, 6 g): 22.8 (10.78) (*p* > 0.05) Effect size = 1.29
Aoe et al., 2014 [38]	β-Glucan	2.9 g	Subjects: 21 (F: 30–49 y) Study design: randomised, not-blinded Washout: 7-days Energy intake interval: 240 min Food matrix: solid (cooked white rice) Control: cooked white rice without fibre	Data not available	Data not available	783.94 (147.47) (*p* = 0.28) Effect size = −0.34	Data not available to calculate effect size
Lumagaet al., 2012 [39]	β-Glucan (viscosity = 55 mPas)	3 g	Subjects: 14 (8M/ 4F:24–39 y) Study design: randomised, single blind Washout: 7-day Measurement of gut hormones: 180 min Energy intake interval: 180 min Satiety: 180 min Food matrix: liquid (250 mL beverages with isocaloric breakfast) Control: beverages without fibre	AUC: (pg·min/mL) (i) GLP-1: 1312.5 (467.71) (*p* > 0.05) Effect size = 0.18 PYY: 13125 (2806.24) (*p* > 0.05) Effect size = 0.13 GIP: 7000 (9354.14) (*p* > 0.05) Effect size = −0.11	Data not available	767.5 (246.2) (*p* = 0.13) Effect size = −0.59	AUC (mm·min) 5400 (748.33) (*p* < 0.05) Effect size = 1.65
	Pectin (viscosity = 90 mPas)	2.5 g		AUC: (pg·min/mL) (ii) GLP-1: 1125(62.5) (*p* > 0.05) Effect size = −0.18 PYY: 135,00 (5612.99) (*p* > 0.05) Effect size = 0.17 GIP: 6000 (3741.66) (*p*> 0.05) Effect size = −0.28	Data not available	871.2 (296.71) (*p* = 0.59) Effect size = −0.20	AUC (mm·min) 6000 (1122.5) (*p* < 0.05) Effect size = 1.94
Wanders et al., 2014 [40]	Pectin (different fibre forms)	10 g (i) bulking (25 kDa)	Subjects: 29 M (18–30 y) Study design: randomised, single blinded Washout: 12-days Gastric emptying rate: 180 min Energy intake interval: 180 min Food matrix: dairy based liquid 150 mL Control: Different fibre formfor EI and GER: dairy based liquid without fibre Different supplementation methods: Gelled pectin in the form of capsule or liquid	Data not available	13C recovery (i) 74.0 (20.4) min (*p* < 0.05) Effect size = 0.19	(i)1058.09 (341.55) (*p* = 0.44) Effect size = −0.21 (bulking)	Data not available
		10 g (ii) viscous (80 kDa)		Data not available	13C recovery (ii) 75.5 (21.0) min (*p* < 0.05) Effect size = 0.2	(ii) 1058.091 (272.28) (*p* = 0.38) Effect size = −0.23 (viscous)	Data not available
		10g (iii) gelled (15 kDa)		Data not available	13C recovery (iii) 82.2 (17.8) min (*p* < 0.05) Effect size = 0.65	(iii) 1024.65 (238.85) (*p* = 0.17) Effect size = −0.36 (gelled)	Data not available
	Different method supplementation (gelled pectin)	10g (iv) capsule (15 kDa)		Data not available	13C recovery (iv) 64.1 (21.9) (*p* < 0.05) Effect size = −0.91	(iv) 955.38 (308.11) (*p* = 0.03) Effect size = −0.42 (capsule vs. liquid)	Data not available
		10g (v) liquid (15 kDa)		Data not available	13C recovery (v) 98.3 (21.1) (*p* < 0.05) Effect size = 0.82		Data not available
Martinelli et al., 2017 [41]	Polydextrose	6 g	Subjects: 25 (19F/ 6M:18–50 y) Study design: randomised, single blinded Washout: 7-days Energy intake interval: 240 min Satiety: 30 to 240 min Food matrix: composite meal (100 g pudding with 200 mL semi-skimmed milk) Control: Pudding without fibre	Data not available	Data not available	1021.27 (356.60) (*p* = 0.53) Effect size = −0.18	iAUC (mm·min) 5166.67 (666.67) (*p* = 0.934) Effect size = −0.27
Astbury et al., 2013 [42]	Polydextrose	(i) 6.3 g	Subjects: 21 (12M/ 9F) Study design: randomised, single blinded Washout: 1-week Energy intake interval: 90 min Food matrix: liquid (chocolate-flavoured liquid, 400 mL) Control: chocolate-flavoured liquid, 400 mL without fibre	Data not available	Data not available	(i) 1206.5 (420.58) (*p* = 0.22) Effect size = −0.38	Data not available
		(ii) 12.5 g		Data not available	Data not available	(ii) 1128.59 (421.58) (*p* = 0.08) Effect size = −0.56	Data not available
		(iii) 25 g		Data not available	Data not available	(iii) 1042.54 (346.10) (*p* = 0.01) Effect size = −0.81	Data not available
Soong et al., 2016 [43]	Polydextrose	12 g (i) low protein	Subjects: 27 (M: 21–40 y) Study design: randomised, single blind Washout: 5-day GLP-1 hormone: 90 min Gastric emptying rate: 90 min Energy intake interval: 180 min Satiety: 75 min Food matrix: solid (soya bean curd) Control: soya bean curd without fibre	GLP-1: (*n*=15) (i) 338.97 (484.63) (*p* < 0.05) Effect size = 0.18	(i) 0.26 (0.14) min (*p* = 0.05) Effect size = −0.47	(i) 790.71 (231.73) (*p* = 0.13) Effect size= −0.42	iAUC (mm·min) (i) 1892.44 (1570.33) (*p* > 0.05) Effect size = −0.05
		12 g (ii) high protein		GLP-1: (*n*=15) (ii) 625.59 (776.77) (*p* < 0.05) Effect size = 0.60	(ii) 0.18 (0.17) min (*p* < 0.05) Effect size = −0.72	(ii) 774.21 (242.86) (*p* = 0.08) Effect size=−0.48	iAUC (mm·min) (ii) 1809.11 (1402.49) (*p* > 0.05) Effect size = −0.11
Hull, et al., 2012 [44]	Polydextrose	(i) 6.25 g	Subjects: 34 (10M/ 24F) Study design: randomized, crossover, single blind Washout: 1-week Energy intake interval: 90 min Food matrix: liquid (drinking yogurt) Control: drinking yogurt with glucose syrup	Data not available	Data not available	(i) 731.36 (228.56) (*p* = 0.61) Effect size = −0.14	Data not available to calculate effect size
		(ii) 12.5 g		Data not available	Data not available	(ii) 711.52 (263.40) (*p* = 0.44) Effect size = −0.21	Data not available to calculate effect size
Juvonenet al., 2009 [45]	β-Glucan	(i) High viscosity (10 g) (>3000 mPas)	Subjects: 20 (16F/ 4M) Study design: randomized, single blinded Washout: > 2-days Measurement of gut hormones: 180 min Gastric emptying rate: 90 min Energy intake interval:180 min Food matrix: liquid (300mL isoenergy and isovolumetric beverage with 200mL water *Low viscosity acts as control	GLP-1 (AUC): (pg·min/mL) (i) 74 (89.44) (ii) 189 (214.66) (*p* = 0.030) Effect size = −0.70 PYY (AUC): (i) 129 (720.01) (ii) 668 (822.87) (*p* = 0.038) Effect size = −0.70 CCK (AUC): *n*=17 (i) 250 (181.42) (ii) 449 (300.99) (*p* = 0.006) Effect size = −1.78	Paracetamol AUC (*n = 10) (i) 14670 (3443.55) (μmol·min/L) (ii) 16340 (5567.81) (μmol·min/L) (*p* = 0.051) Effect size = −0.36	Combined (preload + rest of the day) (i) 1733 (113.05) (ii) 2007.89 (154.16) (*p* = 0.026) Effect size = −2.03	Data not available
		(ii) Low viscosity (10 g) (<250 mPas) *Differed only in viscosity					
Boll et al., 2015 [46]		(i) AXOS, 8.9 g	Subjects: 19 (9M/ 10F: 20–35 y) Study design: randomised, not blinded Washout: 1-week GLP-1: acute (180 min) Food matrix: solid (white wheat bread with 250–300 mL water) Control: white wheat bread without fibre	GLP-1: (i) 1.01 (0.92) (*p* > 0.05) Effect size = 0.10	Data not available	Data not available	Data not available
		(ii) hiAXOS, 18.4 g		GLP-1: (ii) 1.04 (0.92) (*p* > 0.05) Effect size = 0.19	Data not available	Data not available	Data not available
Thazhath et al., 2014 [47]	Guar gum MW: 220 kDa	9 g	Subjects: 12 (6M/ 6F) Study design: randomized, crossover, not blinded Washout: 5-day Gastric emptying rate: every 5 min for 1st hour and every 15 min for further 3 h (240 min) Food matrix: semisolid (mashed potato, 300 mL) Control: semisolid mashed potato 300 mL without fibre	Data not available	13C2 breath used 285 (93.53) (*p* < 0.05) Effect size = 1.02	Data not available	Data not available
Luhovyy et al., 2014 [48]	High-amylose maize	(i) 17.5 g	Subjects: 30M (18–30 y) Study design: randomized, crossover, single blinded Washout: 1-week Energy intake interval: 120 min Food matrix: solid (cookies) Control: cookies without fibre	Data not available	Data not available	(i) 1163.9 (279.34) (*p* = 0.99) Effect size = 0.00	Data not available
		(ii) 31.5 g		Data not available	Data not available	(ii) 1147.5 (310.56) (*p* = 0.83) effect size = −0.05	Data not available
Solah et al., 2014 [48]	PolyGlycopleX (PGX^®^)	(i) 2.5 g	Subjects: 10 (20–29 y) Study design: randomised, single blind Washout: 2 or 3 days Satiety: 120 min Food matrix: liquid (water, 2 × 250 mL) Control: inulin in 500 mL water	Data not available	Data not available	Data not available	iAUC (mm·min) (i) 3501 (2070) (*p* < 0.05) Effect size = 0.56
		(ii) 5 g		Data not available	Data not available	Data not available	(ii) 2937 (1750) mm·min (*p* > 0.05) Effect size = 0.32
		(iii) 7.5 g		Data not available	Data not available	Data not available	(iii)3942 (2250) mm·min (*p* < 0.05) Effect size = 0.74

iAUC, incremental area under curve; AUC, area under curve; *SD*, standard deviation; EI, energy intake; GER, gastric emptying rate; CCK, cholescytokinin; GLP-1, glucagon-like peptide 1; GIP, gastric inhibitory polypeptide; PYY, peptide YY.

**Table 2:** Jadad scores of RCTs (*n* = 17).

Studies		Randomisation	Double-Blinding	Withdrawals and Drop-Outs	Score
Arshad et al., 2016 [35]		1	0	1	2
Wanders et al., 2013 [36]	Study 1	1	0	1	2
	Study 2	1	0	1	2
Rao et al., 2015 [37] *	Study 1	1	1	1	3
	Study 2	1	0	1	2
Aoe et al., 2014 [38]		1	0	1	2
Lumaga et al., 2012 [39]		1	0	1	2
Wanders et al., 2014 [40]		2	0	1	3
Martinelli et al., 2017 [41]		1	0	1	2
Astbury et al., 2013 [42]		2	0	1	3
Soong et al., 2016 [43]		2	0	1	3
Hull, et al., 2012 [44]		1	0	1	2
Juvonen et al., 2009 [45]		1	0	1	2
Boll et al., 2015 [46]		1	0	1	2
Thazhath et al., 2014 [47]		1	0	1	2
Luhovyy et al., 2014 [48]		1	0	1	2
Solah et al., 2014 [48]		2	0	1	3

* only study 1 was double-blinded.
